# *Saccharomyces cerevisiae* as a Tool for Studying Mutations in Nuclear Genes Involved in Diseases Caused by Mitochondrial DNA Instability

**DOI:** 10.3390/genes12121866

**Published:** 2021-11-24

**Authors:** Alexandru Ionut Gilea, Camilla Ceccatelli Berti, Martina Magistrati, Giulia di Punzio, Paola Goffrini, Enrico Baruffini, Cristina Dallabona

**Affiliations:** Department of Chemistry, Life Sciences and Environmental Sustainability, University of Parma, Parco Area delle Scienze 11/A, 43124 Parma, Italy; alexandruionut.gilea@unipr.it (A.I.G.); camilla.ceccatelliberti@unipr.it (C.C.B.); martina.magistrati@unipr.it (M.M.); giulia.dipunzio@unipr.it (G.d.P.); paola.goffrini@unipr.it (P.G.); cristina.dallabona@unipr.it (C.D.)

**Keywords:** mtDNA depletion syndromes, diseases associated with mtDNA deletions, yeast model, *MPV17/SYM1*, *MRM2/MRM2*, *OPA1/MGM1*, *POLG/MIP1*, *RRM2B/RNR2*, *SLC25A4* (*ANT1*)*/AAC2*, drug repurposing

## Abstract

Mitochondrial DNA (mtDNA) maintenance is critical for oxidative phosphorylation (OXPHOS) since some subunits of the respiratory chain complexes are mitochondrially encoded. Pathological mutations in nuclear genes involved in the mtDNA metabolism may result in a quantitative decrease in mtDNA levels, referred to as mtDNA depletion, or in qualitative defects in mtDNA, especially in multiple deletions. Since, in the last decade, most of the novel mutations have been identified through whole-exome sequencing, it is crucial to confirm the pathogenicity by functional analysis in the appropriate model systems. Among these, the yeast *Saccharomyces cerevisiae* has proved to be a good model for studying mutations associated with mtDNA instability. This review focuses on the use of yeast for evaluating the pathogenicity of mutations in six genes, *MPV17/SYM1*, *MRM2/MRM2*, *OPA1/MGM1*, *POLG/MIP1*, *RRM2B/RNR2*, and *SLC25A4/AAC2*, all associated with mtDNA depletion or multiple deletions. We highlight the techniques used to construct a specific model and to measure the mtDNA instability as well as the main results obtained. We then report the contribution that yeast has given in understanding the pathogenic mechanisms of the mutant variants, in finding the genetic suppressors of the mitochondrial defects and in the discovery of molecules able to improve the mtDNA stability.

## 1. Introduction

Mitochondria are cellular organelles present in most eukaryotic organisms, among which include all fungi, plants, and animals. Their main physiological role, albeit not the only one, is the production of most of the cell energy by means of electron transport through the respiratory chain and the oxidative phosphorylation (OXPHOS). The mitochondrial mass inside a cell depends on many factors, including the species and, in multicellular organisms, the cell type, the cell phase, and the energy requirement of the cell.

Mitochondria are semi-autonomous organelles, since they have their own genome, called mitochondrial DNA (mtDNA), which encodes for genes that are fundamental for oxidative phosphorylation. In mammals, mtDNA, which has been discovered in 1963 [1], is circular, approximately 16.5 Kbp long, and contains 37 genes. Thirteen genes encode for subunits of the respiratory chain complexes and of ATP synthase (ND1, ND2, ND3, ND4, ND4L, ND5, and ND6 of complex I; CYB of complex III, CO1, CO2, and CO3 of complex IV; ATP6 and ATP8 of complex V), 2 genes encode for mitochondrial rRNA, and 22 genes for mitochondrial tRNAs. All the approximately 1500–2000 remaining proteins of the mitochondrial proteome, including all the remaining subunits of the complexes involved in OXPHOS, are encoded by nuclear genes, and are imported into mitochondria (reviewed in [2,3]).

In mammals, all the mtDNA present in cells derived from the mtDNA present in the oocyte, and are thus maternally inherited [4]. In addition, excluding the early divisions during embryonic development, mtDNA is synthesized during the whole cell cycle. Indeed, each cell contains several molecules of mtDNA, whose number depends on the cell type and the energetic requirement. Cells that contain most copies of mtDNA are neurons, muscle fibers, hepatocytes, and oocytes, where the mtDNA copy number is equal to thousands or tens of thousands (reviewed in [5,6,7]).

MtDNA molecules are not naked but organized in DNA–protein complexes called nucleoids. Several nucleoids are present inside mitochondria, and each of them contains in general a single mtDNA molecule. Nucleoids, which are anchored to the inner mitochondrial membrane (IMM), form replication units that are autonomous in mtDNA replication and segregation [8,9,10]. According to the most accepted model, mtDNA is replicated in mammals through the strand displacement model [11]. DNA synthesis is continuous on both strands, called H and L; however, the replication is asymmetric, since it starts from two different dedicated replication origins (O_H_ and O_L_) and at different times (reviewed in [12]). Several proteins are involved in the mtDNA replication, and mutations in most of nuclear genes encoding for these proteins are associated with mitochondrial diseases characterized as secondary mutations by mtDNA deletions or depletion. 

If mutations occur, mutated mtDNA molecules are produced. If two mtDNA molecules with a different sequence exist inside a tissue, for example, a wild-type one and a mutant one, either homoplasmic or heteroplasmic conditions are possible. In the former case, each cell contains a single type of mtDNA, whereas, in the latter case, each cell contains both mutant and wild-type copies of mtDNA. In most patients affected by mutations in mtDNA, including secondary deletions caused by nuclear mutations, heteroplasmy is present. These deletions can be considered recessive [13], and pathology occurs only when the number of mutant copies is above a threshold level, whose percentage depends on the mutation and on the affected tissues [14].

Mutations in human nuclear genes involved in mtDNA replication, integrity, maintenance, and segregation are associated with a plethora of mitochondrial diseases associated with mtDNA depletion or multiple deletions. In these disorders, the primary cause is one or more mutations in nuclear genes which, in turn, result in secondary qualitative (multiple mtDNA deletions) or quantitative (mtDNA depletion) mtDNA defects (reviewed in [2,3,5,15]). Depending on the gene and on the mutation, the disorder can be inherited by means of either recessive or dominant inheritance. In the latter case, the dominance can be due to a loss of function, i.e., to haploinsufficiency, to a gain-of-function, or to negative dominance. Whereas mutations in some genes are associated primarily with mtDNA depletion and mutations in other genes primarily to multiple mtDNA deletions, mutations in most genes affecting mtDNA maintenance can be associated with either depletion or multiple deletions. Genes associated with such pathologies are reported in Table 1. 

Since the discovery that mitochondrial diseases associated with mtDNA depletion and/or multiple deletions can be caused by mutations in nuclear genes, the yeast *Saccharomyces cerevisiae* has been used to model these mutations with different aims. Recently, novel mutations in genes previously associated with mitochondrial diseases and mutations in novel genes have been found in patients through whole-exome sequencing (WES) or whole-genome sequencing (WGS). When mutations are identified in a patient through these analyses, often the familiar history is not known, therefore a model system can be useful to “validate” mutations, i.e., to confirm that the variant is the cause of the disorder and not a single nucleotide polymorphism (SNP). In addition, as detailed below, model systems can be useful for understanding the molecular mechanisms through which the mutations exert their pathological effects as well as to find genetic methods or drugs able to rescue the detrimental effects.

Yeast mtDNA is longer compared to its mammalian counterpart: depending on the strain, the length can be 68 Kbp (in short strains) to 86 Kbp (in long strains). The mitochondrial genome currently used as a reference is that sequenced by Foury and coauthors from strain FY1679, isogenic to the reference strain S288C [50]. The yeast mitochondrial genome contains seven genes encoding for subunits of the respiratory complexes (*COB* for complex III; *COX1*, *COX2*, *COX3* for complex IV; *ATP6*, *ATP8*, and *ATP9* for complex V), one gene, *VAR1*, encoding for a subunit of the mitochondrial ribosome, two genes for the 15S rRNA and for the 21S rRNA, 24 genes for tRNAs, and one gene, *RPM1*, encoding an RNA subunit of the RNase P, which is involved in the processing of the mitochondrial pre-tRNAs. In addition, several genes encoding for maturases, endonucleases, and a reverse transcriptase are present, and some of them are located inside the introns of *COX1*, *COB*, and 21S rRNA genes, however their role has not been fully characterized (www.yeastgenome.org, accessed on 19 November 2021).

Yeast mtDNA is rich in AT-segments, especially in the intergenic regions, with small regions rich in GC-segments, among which four copies of regions called *ori* initially associate with mtDNA replication [51,52]. Until now, several hypotheses have been proposed in order to explain the mechanisms of mtDNA replication: according to the most recent findings, *S. cerevisiae* mtDNA should be replicated through a rolling circle mechanism in which the leading strand is primed by recombinational structures, and which results in the coexistence of circular molecules and linear concatemers of DNA [53,54,55,56] (reviewed in [57,58]).

As for its human counterpart, yeast cells contain several copies of mtDNA molecules. Although the exact number depends on several conditions, such as the carbon source added to the growth medium, the growth temperature, and the haploid/diploid status of yeast, it has been estimated that each cell contains 10–50 copies per nuclear genome to 50–200 ones [59,60,61]. As in human mitochondria, yeast mtDNA is packaged into protein–DNA complexes, called nucleoids, which are anchored to the IMM [62,63,64]. Several proteins are present in the nucleoids, among which proteins involved in replication, transcription, repair and recombination, heat shock proteins, and some enzymes of the Krebs cycle [60,62,64,65,66,67].

One of the most important characteristics of *S. cerevisiae* as a model to study mtDNA instability is its *petite* positivity, i.e., yeast can survive without mtDNA or with long deletions in it. In this case, energy is produced through alcoholic fermentation, provided that a fermentable carbon source is added in the medium. Due to the limited quantity of ATP produced through alcoholic fermentation and the inability to utilize the ethanol released in the medium, the colonies deriving from cells harboring these mutations have a smaller size and are called “*petite*”. The *petite* phenotype can be caused by mutations in nuclear genes involved in the maintenance of the mtDNA integrity and are inherited either in a Mendelian way (*pet* mutants) [68] or directly by mtDNA mutations (cytoplasmic *petite* mutants) [69]. Cytoplasmic *petite* mutants, called more simply “*petites*”, arise spontaneously at high frequency (around 1–10%, depending on the strain and on the growth condition), and can be devoid of mtDNA (*rho*^0^ cells) or carry long and multiple deletions of mtDNA (*rho^−^* cells); in *rho^−^* cells, the mtDNA often contains several repeats of the same sequence. [70,71,72]. Cells containing the whole mtDNA are called *rho^+^*. It has been postulated that *rho^−^* cells are generated mainly by homologous recombination, which is highly active in mitochondria, between imperfect repeats [73,74]. Besides, most *rho^−^* mtDNA genomes are unstable and can result in the loss of mtDNA, making the cell *rho*^0^. Mutations in nuclear genes can affect the *rho* status of the cells and influence the frequency of *petite* mutants. Among these genes, there are those involved in the replication, recombination, and repair of the mtDNA, but also in the maintenance, in the integrity, and in the inheritance of the mitochondrial genome (reviewed in [75]).

Contrary to mammals, heteroplasmy is just a transient condition in yeast. If two types of DNA molecules are present in a cell, for example, a *rho^+^* molecule and a *rho^−^* molecule, after a few generations, yeast become homoplasmic, i.e., there are two populations of cells, each with only a single type of mtDNA genome [70,76,77]. Although the exact mechanisms leading to homoplasmy in few generations are not fully understood, it has been hypothesized that several pathways could be involved, such as the positioning of the nucleoids in a different part of the cells, the transport of mitochondria in the buds, the concatemeric formation of mtDNA, and the asymmetric inheritance of mtDNA [55,78,79,80,81,82].

In this review, we focus on the use of yeast for studying pathological mutations in nuclear genes associated with mtDNA instability, underlying the methodologies used to construct the models and to study the phenotypes associated with mtDNA instability and the main findings to which yeast has contributed [50,51,52,53,54,55,56,57,58,59,60,61,62,63,64,65,66,67,68,69,71,72,74,75,76,77,78,79,80,81,82,83,84].

## 2. Creation of the Model and Techniques Used to Measure Instability of mtDNA

### 2.1. Construction of the Model Systems

As reported in Table 1, several human genes associated with mtDNA depletion and/or deletion pathologies are present and conserved in yeast. To evaluate the consequence of a given mutation, it must be considered whether to use, as a wild-type reference, the *S. cerevisiae* gene (homologous complementation), the human cDNA (heterologous complementation), or a chimeric construct between the two genes (chimeric complementation) [85]. Each of these complementation approaches has its respective pros and cons which should be considered. The use of heterologous complementation allows for the studying of any mutation under analysis, since the human gene is directly introduced in yeast. In addition, if a detrimental effect is observed, the allele is very likely pathogenic; on the contrary, if no effect is observed, the allele is likely either not pathogenic or hypomorphic, although it cannot be excluded that, in humans, the gene has a second function which cannot be studied in yeast. Despite these advantages, heterologous complementation is only possible in the case that the human cDNA complements the deletion of its yeast counterpart. Moreover, the complementation degree should be sufficient to evaluate the possible defective phenotypes associated with the mutant alleles, and the gene expression should not be too high to hide the detrimental effect of mutant alleles. If complementation does not occur, chimeric complementation can be exploited. As has been reported, the mitochondrial targeting sequence (MTS) can be different between yeast and animals [86]; in some cases, it is sufficient to replace a human MTS with a yeast one. Both the MTS of the corresponding yeast gene and a generic MTS can be used. After the processing of the MTS, the protein present in the mitochondria is equal to the human one. In other cases, it is necessary to replace a longer region of the human protein with that of yeast to allow complementation, creating a true chimeric protein. If chimeric complementation does not work, homologous complementation must be used. The advantage of using the yeast gene is that it can be cloned under its natural promoter, avoiding effects due to non-physiological expression. However, only amino acids which are conserved or lie in a conserved stretch between the human protein and the yeast protein can be studied. In the former case, the gene can be mutagenized to introduce the mutation, resulting in the amino acid substitution. In the latter case, if the amino acid lies in a conserved region that aligns unambiguously with its human counterpart, the yeast gene must at first be humanized, i.e., the amino acid present in the human wild-type gene must be introduced and, once it has been established that the “humanization” has no or minimal effects on the phenotype, the alleged pathogenic amino acidic variant can be introduced. The use of the homologous complementation is based on the hypothesis that, if an amino acid is conserved during evolution, or lies in a conserved region, it should carry out the same function in all the orthologous proteins. For this reason, if the substitution in the yeast gene results in an affected phenotype, it is very likely that the human substitution is also pathological; on the contrary, if no detrimental phenotype is observed in yeast, it cannot be excluded that it is not pathological in humans.

Although, in the case of homologous complementation, it is possible to introduce the mutation on the yeast genomic locus through specific techniques such as *Delitto Perfetto* or the CRISPR/Cas9 (reviewed in [87]); in most cases, the mutant strain is obtained by one-step gene disruption of the gene under analysis [88] and the transformation with wild-type or mutant alleles introduced in a cloning centromeric plasmid under its natural promoter. On the contrary, in the case of heterologous complementation, the human cDNA must be cloned in an expression vector, which can be centromeric (single copy) or episomal (multicopy), under a specific yeast promoter, and is then introduced in a null yeast mutant. The promoter can be either that of the orthologous yeast gene or, more often, an ad hoc yeast promoter. In general, a constitutive promoter, such as *CYC1* [89], *PGK*1 [90], *ADH1* [91]; an inducible promoter, such as *CUP1* [92] or *GAL1* [93]; or a highly regulatable promoter, such as TETOff or TETOn [89,94], are used. Cloning under such promoters can require a consensus sequence that optimizes the start of translation (Kozak sequence), which for yeast is (A/T)A(A/C)A(A/C)A, inserted just upstream of the start codon [95].

If the gene under analysis is fundamental for the maintenance of the mtDNA, its deletion in a haploid strain leads to mtDNA loss, thus resulting in a *rho*^0^ strain, in which reintroduction of the wild-type gene does not recover the presence of mtDNA. To overcome this problem, several techniques can be used to maintain the mtDNA before the introduction of the mutant allele, as reviewed in [85,96]. The most used technique is the plasmid shuffling strategy, wherein one-step gene disruption is performed in an *ura3* strain previously transformed with the yeast wild-type gene cloned in a centromeric plasmid harboring *URA3* as a selection marker. After the disruption of the chromosomal gene, the strain is transformed with the mutant allele cloned in a plasmid harboring a different selectable marker. Finally, the double transformant strain is treated with 5-fluoroorotic acid (5-FOA), a drug that is toxic for *URA3* strains. After this treatment, only strains that have lost the *URA3* plasmid and that contain the plasmid harboring the mutant allele can grow.

### 2.2. Evaluation of mtDNA Instability

Thanks to its *petite* positivity, the effects of nuclear mutant alleles on mtDNA stability can be measured through the determination of the *petite* frequency, which is the ratio between the number of *petite* colonies and the number of total colonies deriving from a population of cells. The higher the detrimental effects of the mutation on the maintenance of the integrity and on the stability of the mtDNA are, the higher the frequency of *petites* is. The *petite* frequency of a strain depends on two factors: the strain background, including the mutant allele under investigation, which affects the onset of *petites* per generation, and an extrinsic factor, which depends on the growth conditions and can influence both the onset of *petite* cells and the growth rates of *rho^+^* vs. *petite* cells [75]. In order to minimize the effects that interfere with the onset of the *petites*, a comparison between strains harboring the wild-type and the mutant alleles must be performed in the same genetic background, and in the same growth conditions. Although three main methods can be used to measure *petite* frequency (described in [83]), all the methods are based on a pre-growth in a medium supplemented with a non-fermentable carbon source, such as ethanol or glycerol, in order to minimize the initial percentage of *petites*, which cannot grow in such conditions. After this counterselection step, the cells are grown in liquid or, through replica plating, in solid medium in the presence of a fermentable carbon source, such as glucose, in order to allow the onset and the growth of *petite* cells. The growth is performed for several generations (at least 10–15), in order to reach a *petite* frequency that is constant and mainly due to the mutation present in the strain. Both these “bulk” methods offer the advantage that the onset of *petites* occurs independently several times, resulting in a rather constant frequency. Alternatively, a third method is based on measuring the *petite* frequency of cells deriving from single colonies; since, in this case, the number of *petites* in each colony is strongly influenced by the time of the onset of the first *petite* cell, the *petite* frequency of each colony is highly variable and thus the results must be analyzed as in a fluctuation test based on the median [84].

In order to evaluate whether a mutant allele results primarily either in the loss of mtDNA or in the onset of deletions of mtDNA, it is possible to discriminate whether the *petites* are *rho*^0^ or *rho^−^* cells, which mimics a situation either of depletion or of heteroplasmic mtDNA deletions, respectively, in human cells. Three main methods allow for the distinguishing of *rho^−^* and *rho*^0^ colonies. The first is based on crossing *petite* colonies of the mutant strain with tester strains of opposite mating type, which are harbor *mit^−^* in a single point mutation in a mitochondrial gene encoding for a respiratory complex subunit and thus are respiratory deficient (RD). Since recombination in yeast mitochondria occurs at a high rate, after crossing a *petite* colony with a battery of tester *mit^−^* strains, which should include at least mutants in *COB*, *COX2* e *COX3*, if at least one of the diploid strains is respiratory proficient (RP), it means that the tested colony retained an mtDNA fragment encompassing the *mit^−^* mutation of the tester strain and is thus *rho^−^*. This is due to the fact that most of the *rho^−^* mutants retain at least one gene among *COB*, *COX2*, and *COX3* [97,98]. The pro of this technique is that several colonies can be analyzed at the same time, making more powerful statistical tests possible; however, this technique can be used only for haploid strains, and some *rho^−^* colonies can be categorized erroneously as *rho*^0^ if they do not contain any of the abovementioned genes. The second method is based on the extraction of mtDNA from single *petite* colonies, digestion with a restriction endonuclease and Southern Blot using an *ori* fragment as a probe, since at least one *ori* sequence is maintained in most of the spontaneous *rho^−^* cells [70]. The pro of this technique is that almost all the *rho^−^* colonies are identified correctly; however, the technique is time-consuming and can be applied only in a limited number of clones. The third method is based on the staining of mtDNA with a specific probe, such as 4′,6-Diamidine-2′-phenylindole dihydrochloride (DAPI), which, in yeast, binds both nuclear DNA and mtDNA, and on the subsequent visualization of cells through a confocal or an epifluorescence microscope. The mtDNA appears as small spots in the periphery of the cell, which are not present in *rho*^0^ cells. The pros and cons of this method are similar to those of the previous method.

### 2.3. Evaluation of the mtDNA Levels

Independent from the increase in the *petite* frequency, the mutant alleles of genes involved in the maintenance of the mtDNA can result in a decrease in the mtDNA levels. In this case, the strain is RP, but the mtDNA copy number is decreased. The mtDNA levels can be measured through quantitative PCR (qPCR), amplifying a region of one of the mitochondrial protein genes as a target and a region of the nuclear DNA, such as *ACT1*, as a reference [99]. For example, mtDNA levels can be measured using oligos amplifying a region of *COX1*, such as qCOX1Fw (CTACAGATACAGCATTTCCAAGA) and qCOX1Rv (GTGCCTGAATAGATGATAATGGT) [100]. A comparison of the mtDNA/nuclear DNA ratio between strains transformed with the wild-type allele and with the mutant allele allows for the evaluating of whether the mutation is associated with a decrease in the mtDNA levels. It must be underlined that, unless the mutant is *petite*-negative, such as the *aac2Δ* strain, this analysis should not be performed in the presence of a fermentable carbon source, especially if the mutant allele is associated with an increase in the *petite* frequency. In such conditions, *rho^−^* cells can be present, invalidating the results. As a matter of fact, several *rho^−^* cells contain an mtDNA fragment repeated several times, and thus the mtDNA levels could be erroneously misestimated. The analysis should thus be performed by growing the strain in a nonfermentable carbon source, where only *rho^+^* strains with intact mtDNA molecules can grow.

## 3. Genes Studied in Yeast

### 3.1. MPV17/SYM1

An intriguing protein necessary for mtDNA maintenance is MPV17, whose function was puzzling and elusive for a long time and is still not yet completely understood. Originally, MPV17 was considered as a peroxisomal membrane protein [101,102], however, in 2006, it was clearly demonstrated that it is mitochondrially localized [16].

To date, it is known that the human *MPV17* gene encodes a small hydrophobic protein of 176 amino acids embedded in the IMM and characterized by four predicted hydrophobic transmembrane domains and short hydrophilic stretches in the intermembrane space (IMS) and matrix regions [16,103].

Mutations in *MPV17* were initially identified in three families with hepatocerebral mtDNA depletion syndrome [16] and in probands with Navajo neurohepatopathy, an autosomal recessive multisystem disorder [104]. So far, 41 pathogenic variants have been reported in the *MPV17* gene on The Human Gene Mutation Database (HGMD) (http://www.hgmd.cf.ac.uk/ac/index.php, accessed on 20 October 2021). Although the clinical presentations associated with *MPV17* mutations are highly variable, hepatopathy and neurologic abnormalities are the most recurrent clinical features [105]. Symptoms generally occur in the first months of life or in infancy, although cases of adult-onset neuropathy and leukoencephalopathy or progressive external ophthalmoplegia (PEO), characterized by multiple mitochondrial DNA deletions rather than depletion, have been reported [106,107,108].

Based on sequence homology (48% similarity and 32% identity), as well as the similar organization of transmembrane domains, the ortholog of *MPV17* in the yeast *S. cerevisiae* is *SYM1* (Stress-inducible Yeast Mpv17), a gene identified in 2004 which is induced by heat stress and necessary for growth on ethanol at 37 °C [109]. Furthermore, the expression of human *MPV17* in *sym1Δ* null mutant complemented the phenotype, demonstrating functional homology [16,109].

Given the functional conservation, yeast was first used to demonstrate a causative role between the alleged pathological mutation identified for the first time in *MPV17* and the disease [16]. The temperature-sensitive OXPHOS phenotype of the *sym1Δ* yeast strain was rescued by re-expressing the wild-type *SYM1* gene, whereas very limited correction was observed by expressing *sym1^R51Q^*, a variant harboring the mutation equivalent to human R50Q, and no correction was obtained with *sym1^R51W^* and *sym1^N172K^*, the equivalent to human R50W and N166K, thus validating the pathogenic significance of the human mutations [16,17]. In agreement with this observation, the human R50Q, the equivalent to the yeast R51Q mutation, is associated with a milder phenotype [16]. Four additional missense mutations (G24W, P104L, A168D, and S176F, the equivalent to human mutations G24W, P98L, A162D, and S170F, respectively), localized in different protein domains, were studied in yeast, demonstrating deleterious effects for all mutations regarding OXPHOS metabolism and mtDNA stability, measured as the frequency of *petite* colonies [17]. To elucidate the molecular consequences of the mutations, protein stability and localization were assessed. The results obtained showed that all the Sym1 mutant proteins correctly localize into the mitochondria, indicating that no mutation compromises the mitochondrial target sequence, which is still unknown, and suggest that protein instability is the main molecular mechanism underlying the pathology for mutation G24W (hG24W) [17]. Studies based on blue native gel electrophoresis have demonstrated that Sym1 takes part in vivo within a high molecular weight complex, whose composition is still unknown [110]. This offered another hint for investigation; in fact, it was suggested that, for most of the mutations studied in yeast, the pathogenicity is related to the inability to form a fully assembled functional complex [17]. Notably, in both cell cultures and mouse tissues, MPV17 is part of a high molecular weight complex of unknown composition, which is essential for mtDNA maintenance in the liver of an *MPV17* knockout (KO) mouse model [111].

Beside the validation and the comprehension of the molecular mechanisms underlying the disease, yeast was also used to attempt to elucidate Sym1 protein function. In addition to the role of oxidative growth and of mtDNA stability in stressing conditions, it was demonstrated that Sym1 is necessary for glycogen storage and mitochondrial morphology [110]. In fact, ultrastructural analyses have shown that the mitochondria of the null mutant are spherical with flattened or absent mitochondrial *cristae* and some mitochondria show electron-dense bodies [110]. Similar results were obtained on mitochondria from *Mpv17^−/−^* KO mouse [112] and zebrafish [113]. Furthermore, several observations point out a defective Krebs cycle in the *sym1Δ* strain, confirmed by a reduction in succinate dehydrogenase (SDH) activity. This finding is in agreement with the reduction in glycogen accumulation, a process dependent on gluconeogenesis and therefore regulated by the anaplerotic flow of the intermediates of the Krebs cycle from the mitochondria to the cytosol [110]. Interestingly, patients with *MPV17* mutations suffer from severe, sometimes fatal, hypoglycemic crises [16,114]. The data obtained in yeast suggest that these are due to a glycogen deficiency in the liver, providing a possible explanation of this clinical phenotype [110].

The reconstitution of purified Sym1 into lipid bilayers and electrophysiological measurements demonstrated that Sym1 forms a membrane pore in the IMM, whose diameter is large enough to enable the passage of metabolites, the nature of which is not yet known [115]. Similar results were obtained with recombinant MPV17, revealing that it forms a non-selective channel with a pore diameter of 1.8 nm and suggesting the role of MPV17 as a Δψm-modulating channel that contributes to mitochondrial homeostasis [116]. However, the physiological role of the channel and the nature of the cargo remain elusive.

Although biochemical functions of Sym1/MPV17 remain mainly unknown, it appears to be essential for mtDNA copy number maintenance, since the loss of the function of this protein causes mtDNA depletion in patients [16] and in *MPV17* KO mice [112], and mtDNA instability in *S. cerevisiae* [16,17,110]. However, the role of MPV17 in mtDNA maintenance is not yet completely understood and several hypotheses have been proposed. The enhanced reactive oxygen species (ROS) production observed in the glomeruli of *Mpv17* KO mice suggests an involvement of MPV17 in the regulation of ROS levels [117], even if it is not clear whether the ROS increase is a consequence of an impaired OXPHOS process resulting from the reduction in mitochondrial DNA content or the cause of the mtDNA damage [118]. A decrease in mitochondrial deoxynucleoside triphosphate (dNTP) pool, observed in the liver mitochondria of rat tissues and fibroblasts derived from patients with mutations in the *MPV17* gene, and the demonstration that supplementation of dNTPs prevents and rescues mtDNA depletion in patients’ fibroblasts, strongly suggest that the insufficient availability of mitochondrial dNTPs is the principal cause of mtDNA depletion [119]. At a molecular level, it seems that MPV17 supports the mitochondrial purine salvage pathway, since a decreased expression of enzymes involved in this pathway was observed in the *Mpv17* KO mouse and in patient-derived fibroblasts [119]. The hypothesis that MPV17 may be involved in mitochondrial nucleotide metabolism is also supported by the observation that the deficiency of *MPV17* orthologous gene in zebrafish results in a strong reduction in pigment cell iridophores, mainly constituted by guanine [120]. It has been proposed that the lack of MPV17 leads to a reduction in the uptake of guanosine or its phosphate derivatives, resulting in mitochondrial dysfunction and in iridophore death. Moreover, iridophore and melanophore loss in zebrafish embryos can be caused by the chemical inhibition of pyrimidine de novo synthesis [121]. Interestingly, it has been demonstrated that supplementation with dNTPs and pyrimidine precursors as orotic acid leads to a significant increase in both iridophore number and mtDNA content in *mpv17^−/−^* zebrafish mutants, thus linking the loss of MPV17 to pyrimidine de novo synthesis [113]. Furthermore, MPV17 deficiency in HeLa cells has been shown to be associated with a reduction in folate levels and with an increase in the uracil level, a marker of impaired deoxythymidine monophosphate (dTMP) synthesis, without compromising either de novo or salvage pathway. This suggests that MPV17 can provide another dTMP source and prevents uracil misincorporation in mtDNA [122], which could lead to DNA strand breaks and genome instability [123]. On the other hand, in *S. cerevisiae*, Sym1 has been related to a homeostatic control of tricarboxylic acid cycle (TCA) intermediates, such as oxalacetate and α-ketoglutarate [110]. 

Very recently, it was shown that the yeast mitochondria of *sym1Δ* mutant displayed a significant decrease in the levels of all the dNTPs, suggesting that, as in the *Mpv17^−/−^* mouse and in *MPV17*-deficient human fibroblasts, the instability of mtDNA in *sym1∆* yeast mitochondria is associated with a decrease in dNTPs levels, providing strong evidence that the cause of the mtDNA deletion/depletion in Sym1 deficient cells is a shortage of precursors for DNA synthesis [40]. Furthermore, it was demonstrated that the nucleotide reduction is not limited to the mitochondrial compartment but is instead extended to the entire cell compartment. The whole-cell dNTP pool decrease could be due to a reduced ATP production caused by the OXPHOS impairment; however, OXPHOS defect and mtDNA instability occur independently in *sym1Δ* yeast cells [110]. Another tempting explanation links this dNTP shortage to the Sym1 proposed function, as TCA cycle intermediates homeostatic control [40]. Indeed, it was previously reported that the OXPHOS defect of *sym1Δ* strain was rescued by the overexpression of mitochondrial transporters of TCA intermediates (*YMC1* and *ODC1*), or by the supplementation of different amino acids (glutamate, aspartate, glutamine, or asparagine) produced from TCA intermediates and all precursors of nucleotides synthesis [110]. Finally, as yeast lacks a deoxynucleotide salvage pathway, the mitochondrial dNTP pool is exclusively dependent on cytosolic dNTPs transport; as such, the reduction in the cytosolic dNTP pool could be reflected in a decrease in mitochondrial nucleotides.

After 15 years of intense studies, many steps forward have been made to understand the role of MPV17/Sym1 in the cell: it has been shown that it forms a non-selective channel, and it has been clearly demonstrated that it is involved in maintaining a correct level of dNTPs in mitochondria and, consequently, in the stability of mtDNA; however, the nature of the cargo and the exact function of MPV17/Sym1 remain to be clarified.

### 3.2. MRM2/MRM2

*MRM2* (Mitochondrial rRNA Methyltransferase 2, also known as *RRMJ2* or *FTSJ2*) encodes for a mitochondrial 2’-O-ribose methyltransferase, which is required for a correct maturation of mitochondrial rRNA. In particular, MRM2 mediates the methylation of U(1369) located in the A-loop of the 16S rRNA, the core of the large mitochondrial ribosome subunit [124,125,126]. Recently, a structural approach provided essential insights into the last steps of the large mitoribosomal subunit biogenesis pathway [127]; however, the exact role of the RNA modifications is largely unknown.

U1369 is in the peptidyl transferase center and is implicated in the interaction of the ribosome with a tRNA in the aminoacyl(A)-site. 2′-O-ribose methylation of uridine 1369 was shown to be critical for proper mitochondrial translation and, consequently, for mitochondrial respiratory function [126]. In fact, siRNA knockdown cell lines showed a reduction in mitochondrial protein synthesis flanked by a reduction in the oxygen consumption rate (OCR) [126]. However, further studies are required to elucidate the exact functional role of the U1369 modification. In bacteria, 2′-O-ribose methylation of the A-loop uridine is not only required for proper ribosome biogenesis, but also plays a role in regulating translational accuracy [128]. Interestingly, MRM2 was found to be in the proximity of mtDNA nucleoids in mouse cell-cultures [124].

In 2017, a pathological mutation in *MRM2* was associated with childhood-onset of rapidly progressive encephalomyopathy and stroke-like episodes [18], underling the importance of rRNA methylation for mitoribosomal function. Multiple OXPHOS defects and a decreased mtDNA copy number were detected in muscle homogenate [18]. On the contrary, primary fibroblasts derived from the patient did not recapitulate the mitochondrial phenotypes, possibly due to tissue-specificity, pushing the authors to use the yeast *S. cerevisiae* to study the pathogenicity, thanks to the presence of the ortholog gene *MRM2*. It was previously demonstrated that not only is the human MRM2 highly similar to yeast Mrm2 on the sequence level, but that it also retained a functional homology during ribosome biogenesis [126]. The yeast Mrm2 protein is responsible for the 2′-O-ribose methylation of U2791 in 21S rRNA [129], which is equivalent to human U1369 of 16S [126]. Deletion of the *MRM2* gene led to a thermosensitive oxidative growth defect and rapid loss of mtDNA [129].

Notably, the yeast model expressing the G259R variant, the equivalent to human substitution G189R, showed a significant reduction in respiratory activity at the permissive temperature (28 °C) that was further exacerbated at the stressing temperature (37 °C), thus validating the pathological significance of this mutation [18]. Furthermore, through a radioactively labeled reverse transcriptase primer extension (RT-PEx) reaction analysis, it was shown that the substitution of the conserved residue results in a diminished Um2791 modification in yeast mitochondrial 21S [18], thus providing an insight into the molecular mechanism underlying the dysfunction.

### 3.3. OPA1/MGM1

*OPA1* (Optic Atrophy 1) encodes for a mitochondrial dynamin-like GTPase mainly involved in mitochondrial fusion, *cristae* integrity, mtDNA stability, and copy number maintenance [130,131,132,133]. Beside these roles, OPA1 is also implicated in apoptosis regulation [134,135], in mitochondrial quality control [136,137], and in mitochondrial homeostasis regulation [19,138,139]. 

OPA1 is composed of an N-terminal MTS followed by a transmembrane domain (TM), a coiled-coil domain and three highly conserved dynamin constituents: a GTPase domain, a middle domain, and a coiled-coil GTPase effector domain (GED) [140]. Alternative splicing of exons 4, 4b, and 5b leads to eight OPA1 variants with a tissue-specific pattern of expression [141]. These variants are required to finely tune and to provide flexibility of mitochondrial dynamics under different cellular conditions [140]. The cleavage of the MTS produces long isoforms collectively called long-OPA1 (l-OPA1), which are anchored to the IMM and are essential for mitochondrial fusion and *cristae* organization [142,143]. Through a mechanism known as alternative topogenesis, about half of the long isoforms are then subjected to a proteolytic process in the rhomboid cleavage region (RCR) located before the GTPase domain to generate the short isoforms, called short-OPA1 (s-OPA1), which are devoid of the TM segment [144] and localized in the IMS. In addition, it is known that the short forms interact with some subunits of the mitochondrial contact site and *cristae* junction organization system (MICOS) to maintain the integrity of *cristae* junctions [140,145]. A specific ratio of l- and s-forms (2 long:2 short or 1 long:2 short) is necessary for an efficient mitochondrial fusion; in fact, an unbalancing toward the l-forms leads to an increase in mitochondrial network fragmentation [140]. 

*OPA1* mutations are associated with dominant optic atrophy, one of the most common inherited optic neuropathies, which is characterized by the degeneration of the retinal ganglion cells (RGCs) and by an insidious onset of visual impairment in childhood, with moderate to severe loss of visual acuity [21,146,147,148]. To date, hundreds of pathological mutations have been identified (https://databases.lovd.nl/shared/variants/OPA1/unique, accessed on 20 October 2021) as the cause of Dominant Optic Atrophy (DOA). *OPA1* mutations associated with a DOA cluster mostly in the GTPase domain and are caused mainly by substitutions and by single deletions and insertions generating haploinsufficiency [149,150]. Some DOA patients that harbor missense *OPA1* mutations with a severe dominant-negative effect due to the interference of the mutant variant with the wild-type one display a syndromic form of DOA named DOA plus or DOA+. In these patients, which represent about 30% of those with DOA, optic atrophy in childhood is followed by chronic progressive external ophthalmoplegia (PEO), ataxia, sensorineural deafness, sensory-motor neuropathy, myopathy, and mtDNA multiple deletions in adult life [130,131].

The *OPA1* role in mtDNA maintenance has been clarified thanks to the functional homology with the orthologous gene *MGM1* (Mitochondrial Genome Maintenance) of the yeast *S. cerevisiae*. Mgm1 is a dynamin family member protein that was first discovered in *S. cerevisiae* in a genetic screening for nuclear genes required for the maintenance of mtDNA [151]. Subsequent studies indicated an additional role for Mgm1 in IMM fusion and in the formation and maintenance of *cristae* structure [152]. As for OPA1, Mgm1 exists in two forms: long (l-Mgm1) and short (s-Mgm1). The first is an integral IMM protein that spans the inner membrane, thanks to an N-terminal transmembrane segment (TD), while the second is a short soluble isoform present in the IMS [153]. A 1:1 ratio of l- to s-forms is fundamental for correct mitochondrial morphology and function; in fact, the overexpression of l-Mgm1, increasing this ratio, leads to mtDNA loss and mitochondrial network fragmentation [154]. The *mgm1Δ* mutant strain, which contains highly fragmented mitochondria because of the absence of the fusion machinery, is unable to segregate mtDNA and, after a few generations, loses mtDNA and becomes RD. 

As for many genes causing mtDNA deletions, the use of patients’ fibroblasts for studying mtDNA instability has some limitations, and model systems are needed [155]. Besides mammalian cell models and animal models, in recent years, yeast has been proven to be a useful model for evaluating in short times the pathogenicity and the dominance of novel mutations [155]. Although OPA1 and Mgm1 have a similar predicted structure, the sequence identity is limited to approximately 20%, and *OPA1* cannot complement the deletion of *MGM1*. To overcome this problem, which would prevent the study of most mutations, a chimeric complementation approach was used to study the effect of several pathogenic *OPA1* mutations in yeast [20,21]. Six different chimeras with different portions of Mgm1 and OPA1 regions were constructed, and complementation studies demonstrated that one of the chimeras, named *CHIM3*, was able to partially complement the *mgm1Δ* OXPHOS phenotypes. Furthermore, the aberrant mitochondrial morphology of *mgm1Δ*, due to a mitochondrial fusion deficit and not to the *rho*^0^ condition, was partially rescued when *CHIM3* was expressed [20]. *CHIM3* encodes for the MPS, the TM, and the RCR of Mgm1 fused to the catalytic region of OPA1 (GTPase domain, middle domain, and GTPase effector domain), and was cloned under the TEToff promoter in a single copy vector [20]. A similar construct was used in [21].

To validate *CHIM3* as a suitable model with which to study *OPA1* pathological mutations, three well-known missense mutations were introduced in *CHIM3*: I382M associated to DOA but is also present in healthy subjects and was proposed to be a phenotypic modifier [156,157,158]; R445H associated to DOA plus [159]; and K468E associated to DOA [160]. In agreement with the severe pathological role of R445H and K468E substitutions, the corresponding yeast mutant strains showed a deficient respiratory phenotype and lacked tubular mitochondria. Moreover, the ratio between the s- and the l- forms is altered in both mutant strains, suggesting that the processing of l- and s-forms is impaired and that an alteration of the ratio between the l- and s-OPA1 could also be the cause of the disease. In contrast, the strain carrying *chim3^I382M^* mutant was able to complement the deletion of *MGM1*, showing only a 1.5-fold increase in the *petite* frequency, a slight decrease in oxidative growth, and a 20% reduction in respiratory rate as well as of some respiratory complex activities [20], supporting the hypothesis that I382M acts as phenotypic modifier, contributing to the worsening of the phenotype when in compound with another mutation [157].

Moreover, diploid heteroallelic models, harboring a wild-type allele and a mutant allele, also provide a useful approach for detecting the mechanism of dominance, i.e., loss-of-function (that, in humans, is associated with dominance by haploinsufficiency) or gain-of-function [19,20]. In this regard, heteroallelic strains expressing the I382M or K468E mutations and one copy of wild-type *CHIM3* (*CHIM3/chim3^I382M^* or *CHIM3/chim3^K468E^*) displayed a normal oxidative phenotype as with the homoallelic strain (*CHIM3/CHIM3*), indicating a loss of function mutations, while the heteroallelic strain carrying the R445H mutations (*CHIM3*/*chim3^R445H^*), associated to DOA plus, showed an oxidative growth defect indicative of a dominant negative mutation. Further studies, carried out using these yeast models, revealed that most of the missense mutations associated with DOA or DOA plus in the haploid strain cause the inability to grow on oxidative carbon sources due to the loss of mtDNA, whereas, in the diploid strain, they can cause a reduction in oxidative growth, suggesting that the mutant variants interfere with the activity of wild-type protein and are partially dominant-negative. It has been proposed that the severity of the phenotype as defined by pure DOA or DOA plus depends either on the degree of interference of the mutant protein on wild-type one or other factors such as the co-presence of modifying variants in other genes as well as environmental factors or age/gender of the patients [19]. Finally, the fact that the relative amount of l- and s-Mgm1 was altered in most mutant strains indicates that an increased ratio could be used as an additional indicator of the pathogenicity of the mutations. 

### 3.4. POLG/MIP1

*POLG* encodes for the DNA polymerase γ, the catalytic subunit of the only mitochondrial replicase identified in animal mitochondria [161,162]. This enzyme was identified in 1970 as an RNA-dependent DNA polymerase in human Hela cells and represents only 1–5% of the total cellular DNA polymerase activity [163,164]. It is involved in replication, recombination, and the repair of mtDNA [165,166,167,168,169]. In mammals, the DNA polymerase γ acts as a complex containing three subunits: a catalytic subunit of 140 kDa encoded by *POLG* and two accessory subunits of 55 kDa encoded by *POLG2*, that enhance the processivity of the enzyme [170]. The catalytic subunit is composed of three domains: the N-terminal domain (residues 170-440), with a 3′→5′ exonuclease activity involved in the proofreading activity; the C-terminal domain (residues 440–475 and 785–1239), characterized by polymerase activity responsible of the synthesis of mtDNA and a spacer region (residues 475–785) [169,171,172,173,174]. The spacer region is divided into two subdomains: the former is responsible for the intrinsic processivity whereas the latter, which interacts with the POLG2 subunit, is responsible for the enhancement of the processivity [175,176,177].

To date, more than 300 pathogenic mutations of *POLG* have been reported (http://tools.niehs.nih.gov/polg/, accessed on 20 October 2021), and pathologies caused by these mutations can be divided into two main groups: pathologies associated with mtDNA depletion and pathologies associated with multiple mtDNA deletions. The former leads to diseases with infancy to childhood-onset and is characterized by the involvement of many tissues and organs, whereas the latter leads to diseases with adolescence to adulthood-onset and is characterized by the involvement of a limited number of tissues (reviewed in [178]). Among the diseases characterized by mtDNA depletion, there is the lethal childhood myocerebrohepatopathy (MCHS), which affects infants and is characterized by development delay, myopathy, hepatic failure, pancreatitis, acidosis, and occurs generally in the first months of life [179]; the Alpers–Huttenlocher syndrome (AHS), characterized by childhood-onset, and is responsible for severe encephalopathy with liver failure and intractable epilepsy [180]; a MNGIE-like disease that has a variable onset and is characterized by gastrointestinal dysmotility, ptosis, myopathy, and sensory neuropathy [181]. Other syndromes associated with mtDNA deletions and characterized by epilepsy and ataxia include MERRF, MELAS, MEMSA, SCAE, and SANDO [182,183,184,185]. Adult-onset PEO is the most frequent mitochondrial pathology characterized by multiple mtDNA deletions associated to mutations in *POLG* [22,169]. Whereas all the previous pathologies are inherited through an autosomal recessive inheritance, PEO can be recessive (arPEO) and is characterized by the progressive weakness of the extraocular muscles which determine ptosis and ophthalmoparesis, or dominant (adPEO), and is characterized by axonal neuropathy, ataxia, depression, parkinsonism, and hypogonadism [186]. Most of the substitutions causing adPEO are in the polymerase domain, while recessive mutations are found throughout the whole protein. Altogether, mutations in *POLG* represent the main cause of mitochondrial diseases with Mendelian inheritance [178]. Due to the high frequency of mutations and SNPs in *POLG*, several patients harbor three or more mutations and SNPs, and, for this reason, it is often difficult to understand which mutation(s) are the cause of the pathology and whether the mutation(s) are dominant or recessive.

*MIP1* (MItochondrial Polymerase 1) is the orthologous gene of *POLG* in the yeast *S. cerevisiae* and encodes for a protein of 140 kDa [187]. Mip1 shows a 45% similarity with its human counterpart. However, the similarity is not homogeneously distributed along the protein, but it is higher in the exonuclease and in the polymerase domain [35,187]. Mip1 is also characterized by a C-terminal extension (CTE), which is specific to fungal polymerase γ, and is variable among the species and reaches the maximum length in species of the *Saccharomyces* genus, where it plays a key role in balancing the exonuclease and the polymerase activities [188,189,190].

Yeast has proven to be an excellent genetic system to obtain information and validate in vivo the pathogenicity of *POLG* mutations (reviewed in [187]). Several substitutions found in patients affected by *POLG*-related diseases have been introduced in the corresponding position of Mip1 in a *mip1Δ* strain. Most of the mutations have been studied through homologous complementation and, since the deletion of the chromosomal *MIP1* gene leads to the loss of mtDNA, the introduction of *mip1* mutant alleles has been obtained principally by plasmid shuffling on 5-FOA. A few mutations have been studied through chimeric complementation by replacing *MIP1* with human *POLG* and *POLG2* containing the MTS of Mip1 [35]. Besides the determination of the *petite* frequency, discrimination of the nature of the *petites*, and measurement of the mtDNA levels, yeast has been used to evaluate whether substitutions are associated with an increase in mtDNA point mutations and to specific biochemical defects. Various information has been obtained by using yeast models (reviewed in [187]): (i) The higher the *petite* frequency associated with a *MIP1* mutations is, the higher the *rho*^0^ frequency among the *petite* colonies is, indicating that the most severe mutations result primarily in the loss of mtDNA; (ii) Some mutations increase the frequency of mtDNA point mutations, but this increase does not correlate with the increase in the *petite* frequency, indicating that the mechanisms are different. Thus, the increase in the point mutations does not seem to be involved in the development of the pathology. However, a decrease in the replication fidelity may influence the progression of the disease. Interestingly, several mutations in the exonuclease domain strongly increase the *petite* frequency, indicating that the exonuclease domain interacts with the polymerase domain and is fundamental for a proper replication process and to avoid deletions; (iii) Exposure to the exogenous base-alkylating agent methyl methanesulfonate increased the frequency of mtDNA point mutations when mutations in *MIP1* are present, suggesting that mutagenic molecules may negatively affect the progression of the pathology; (iv) Oxidized bases, which are produced in the mitochondria mainly by the presence of ROS, may play a role in the increase in mtDNA instability when some *mip1* mutant alleles are present. Indeed, in several *mip1* mutant strains, the *petite* frequency is decreased by the treatment of antioxidant molecules such as lipoic acid and MitoQ; (v) When more than two mutations were present in patients, yeast allowed to discriminate which mutations are the cause of the pathology; (vi) Yeast allowed to discriminate whether the heredity of a mutation is recessive or dominant; (vii) Moreover, thanks to the results obtained in yeast, two hypotheses can explain the dominance of some mutations in the polymerase domain. Several mutant variants have a similar DNA binding affinity but have no polymerase activity. The mutant variant binds the DNA with the same affinity, thus blocking the replication and preventing at the same time the binding of the wild-type enzyme. Alternatively, some mutant variants may directly introduce lesions to mtDNA, such as oxidized bases; (viii) It has been found that some SNPs are not neutral, but rather phenotypic modifiers, which can worsen the phenotype associated with a mutation; (ix) Although most of the SNPs are neutral and do not affect the *petite* frequency and the frequency of mtDNA point mutations, mutant variants harboring some of these SNPs are much more sensitive to the nucleoside analogs reverse transcriptase inhibitors (NRTIs) used to treat HIV infection, such as stavudine and zalcitabine, or valproic acid, used to treat epilepsy; (x) Almost all the pathological mutations are associated with an increase in the *petite* frequency. In addition, different mutations have a different severity, and the phenotypic defect on mtDNA stability induced by a mutation grossly parallelizes with the severity of the pathology, making yeast a suitable model for predicting the severity of a pathology; (xi) Mutations can affect the function of Mip1 in several ways: some mutations reduce the protein stability, especially at higher temperatures, other mutations reduce the catalytic activity, the processivity, the DNA binding affinity, the affinity for the incoming dNTPs, or the specific constant, whereas other mutations cause an increase in the ratio between the exonuclease activity and the polymerase activity. On the contrary, the exonuclease activity is only slightly or not at all reduced, even in the case of mutations in the exonuclease domain, indicating that the defects of mtDNA replication are not due to defects of the proofreading activity.

Moreover, it was observed that the overexpression of *RNR1* or the deletion of *SML1*, whose function is reported in Section 3.5, reduces the *petite* frequency in most mutant strains harboring pathological mutations by increasing the concentration of dNTPs [23,27,29,30,191]. The lower the mtDNA instability is, the greater the effect of the *RNR1* overexpression or the *SML1* deletion is, indicating that mutant variants that retain most of their catalytic activity benefit more by the increase in the concentration of the dNTPs. The rescuing activity exerted by the dNTPs observed initially in yeast explains the observation that supplementation to the myotubes of patients harboring mutations in *POLG* with specific concentrations of dNMPs, which are easily converted to dNTPs, lead to an almost complete normalization of the mtDNA levels [192].

The increase in the *petite* frequency and the point mtDNA mutability caused by Mip1 mutations are rescued also by the overexpression of DNA polymerase ζ, whose subunits are encoded by *REV3* and *REV7* genes, and by the deoxycytidyl transferase, encoded by the *REV1* gene. Both enzymes are involved in the error-prone translesion synthesis and localize also in mitochondria both in yeast and, though limited to Rev3, in humans [30,193,194]. It was shown that *MIP1* mutations rescued by DNA polymerase ζ overexpression are not recovered by the treatment with antioxidant molecules and vice versa, suggesting two different mechanisms of rescue. The decrease in *petite* frequency induced by overexpression of DNA polymerase ζ could be due to its capacity to partially replace Mip1 variants which mainly decrease the catalytic activity, whereas the decrease in the mtDNA instability induced by antioxidant drugs could be due to a decrease in the concentration of oxidized bases that can be incorporated by other Mip1 variants.

### 3.5. RRM2B/RNR2

*RRM2B* encodes for the Ribonucleotide Reductase Regulatory TP53 Inducible Subunit M2B, which is able to associate with the large subunit R1 to form an active ribonucleotide reductase complex (RNR), which catalyzes the synthesis of deoxyribonucleoside diphosphates from ribonucleotides diphosphates. Its expression is essential for DNA repair and mtDNA synthesis in postmitotic cells, since, in cycling cells, another small subunit called R2 interacts with R1 to form another type of RNR complex [195]. Due to the key role of the RNR complex in the biosynthesis of dNTPs, the limited availability of DNA building blocks for mtDNA synthesis results in mtDNA a maintenance defect when *RRM2B* is deficient. Since the first identification of an *RRM2B* mutation by Bourdon et al. in 2007, about 40 different mutations have been described until now [196]. The clinical manifestations associated with *RRM2B* mutations are very heterogeneous both in terms of severity of symptoms and the age of onset. Mutations in *RRM2B* can result in mtDNA depletion associated with a severe and childhood-onset multisystemic disease [130,197,198,199,200,201] or in the accumulation of multiple mtDNA deletions associated with a milder adult-onset PEO [202]. In the first case, pediatric patients manifest muscle hypotonia and weakness, often associated with severe respiratory distress; the disease progresses very quickly and causes death in a few months from the onset of symptoms [203]. In the second case, patients are clinically characterized by ptosis and weakness of the extraocular muscles [202].

The disease pathogenesis can be caused by mutations that affect amino acids involved in iron binding (crucial for the catalytic activity of the enzyme) amino acids that are essential for the conformation and stability of the active site, or amino acids that allow the interaction of R2 with an R1 subunit, thus interfering with RNR assembly [197,204,205].

In yeast, the large subunit R1 of RNR is encoded by *RNR1* and *RNR3*, while the small subunit R2 is encoded by *RNR2* and *RNR4*. As in other organisms, RNR is a heterotetramer containing two R1 subunits and two R2 subunits. The main isoform present in yeast is (Rnr1)2-Rnr2-Rnr4; whereas expression of *RNR2–4* is constant during the cell cycle, *RNR1* is upregulated before the S phase [206,207,208,209,210,211]. Rnr1 is thus the limiting factor for the assembly of the RNR complex. In addition, Rnr1 is inhibited by Sml1, a protein that binds Rnr1, blocking its assembly with the R2 subunits [212,213]. Concerning the R2 subunits, Rnr2 has a catalytic role, while Rnr4 folds correctly and stabilizes the radical-storing Rnr2 in an Rnr2-Rnr4 heterodimer which localizes in the nucleus during most of the cell cycle but undergoes cytoplasm redistribution during the S-phase in order to form the full RNR complex [214,215,216,217]. Whereas deletion of *RNR2* makes the strain inviable, the deletion of *RNR1* or *RNR4* makes the strain viable but devoid of mtDNA (www.yeastgenome.org).

Human RRM2B and its yeast orthologue Rnr2 share 55% identity and 78% similarity. In order to create a yeast model useful for the search of suppressors or drugs capable of rescuing the phenotype of mutations in *RNR2*, four mutations, equivalent to human substitutions R41P, I224R, M282I, and L317V were introduced in *RNR2*. All the mutant variants increase both the frequency of *petite* colonies and, among these, the frequency of *rho*^0^ colonies, at 37 °C, although at different extents. For all the mutant strains, the increase in the *petite* frequency was partially rescued by the overexpression of *RNR1* and, to a lesser extent, of *RNR4*. Combined with the fact that mutant strains are not fully devoid of mtDNA, and thus Rnr2 mutant variants retain part of their catalytic activity, this observation suggests that an increase in the RNR levels and/or its stabilization could improve the synthesis of the dNTPs, despite the detrimental substitution present in Rnr2 (Baruffini and Dallabona, personal observations).

The most detrimental mutation was L362V, equivalent to human L317V, which increased the *petite* frequency to ~60% [40]. The mutant strain harboring this mutation was used to evaluate the capability of some drugs to reduce the mtDNA instability, as reported in Section 4.

### 3.6. SLC25A4 (ANT1)/AAC2

*ANT1* (Adenine Nucleotide Translocase), also known as *SLC25A4*, encodes for an ADP/ATP transporter, one of the most abundant proteins of IMM. Although the primary function of ANT1 is fully understood, the link with mtDNA instability is highly debated and far from being clarified.

In humans, there are four ANT isoforms with no functional difference but differential tissue expression [218,219]. *ANT1* is highly expressed in post-mitotic cells and encodes for the most abundant isoform in heart and muscle [220]; *ANT2* (*SLC25A5*) is expressed at a low level in differentiated tissues and is particularly abundant in proliferative, undifferentiated cells [221]; *ANT3* (*SLC25A6*) is ubiquitously expressed at variable levels depending on oxidative metabolism; *ANT4* is exclusively expressed in liver, testis, and brain [219]. 

ANT1 belongs to the large family of mitochondrial carriers [222,223], presenting a six alpha-helices transmembrane domain that forms a nucleotide translocation channel [224]. Although ANT1 has been thought to function as homodimers for decades [225,226,227], more recent observations have challenged this point of view and a monomeric functional unit was proposed [228,229,230]. Its primary function is to mediate the 1:1 exchange of ATP and ADP across the membrane, importing cytosolic ADP into the mitochondrial matrix to fuel the ATP production by ATP synthase (Complex V) and exporting to the cytosol the ATP produced by the OXPHOS process and is necessary to support all cellular activities. However, in vitro experiments show that this direction is achieved when cells possess a normal membrane potential that drives productive transport (ATPout, ADPin). If the membrane potential is altered, ANT can mediate non-productive ADP/ADP or ATP/ATP exchange or counterproductive (ATPin, ADPout) transport [231]. 

However, the role of ANT is not limited to ADP/ATP transport; in fact, it has a role in the regulation of the mitochondrial permeability transition pore [232,233] and mitochondria-mediated apoptosis [234], in mitophagy [235], and in proton loss across the IMM, thus mildly uncoupling the membrane and avoiding the hyperpolarization and overproduction of ROS [236].

The first disease-linked substitution in the *ANT1* gene (A114P) was identified more than two decades ago [47], associated with adPEO. Since then, a total of ten missense mutations have been identified in *ANT1*: five (A90D, L98P, D104G, A114P, and V289M) are dominant and were found in patients affected by adPEO, clinically characterized by ptosis and impairment of eye movements [47,237,238,239,240]; two (A123D and R236P) are recessive loss-of-function mutations and have been found in subjects affected by mitochondrial myopathy and cardiomyopathy [41,241]; three are de novo dominant mutations associated with severe non-adPEO disease (R80H and R235G) [49] or with a mild myopathy (K33Q) (King et al., 2018). Despite the wide spectrum of clinical presentations, all patients share a molecular feature, i.e., mtDNA defects in affected tissues. Patients affected by adPEO and patients carrying homozygous recessive mutations present an accumulation of multiple mtDNA deletions whereas patients carrying the de novo mutations show mtDNA depletion. 

The assessment of the impact of *ANT1* dominant mutations in mammals is hindered by the absence of suitable models. In fact, the most used human cell lines, fibroblasts, express the *ANT1* gene at a very low level, and its exogenous expression induces apoptotic cell death [47,242]. Contrariwise, mouse myotubes express naturally high levels of *ANT1*, therefore it was possible to exogenously express mutant alleles. This model was used to assess the effect of some dominant mutations on ANT1 transport activity, demonstrating a decreased ADP/ATP exchange function and abnormal translocator reversal potential; however, no mtDNA deletions or depletion were observed [243]. 

The *ANT1* gene is highly conserved in all eukaryotic organisms, including the yeast *S. cerevisiae*. In yeast three genes coding for the mitochondrial ATP/ADP carrier (*AAC1*, *AAC2*, *AAC3*) have been identified [244,245,246,247], and, among these, *AAC2*, encoding the major isoform of the translocator and the only one required for respiratory growth [245], is considered the yeast ortholog of human *ANT1*. Worth considering is that the *aac2Δ* null mutant is *petite*-negative [248], i.e., it is not viable in the absence of mtDNA. Contrariwise to *rho^+^* respiring cells, in which mitochondrial membrane potential is generated by respiratory complexes proton pumping, in cells lacking mtDNA (*rho*^0^ cells) in which respiration is impaired, a minimal membrane potential is maintained by the electrogenic exchange of cytosolic ATP (containing four negative charges) for mitochondrial ADP (containing three negative charges) by means of Aac2 reversing the transport direction [249,250]. Even if respiration and mitochondrial DNA are dispensable in *S. cerevisiae*, the mitochondrial membrane potential is essential for viability and therefore the absence of both Aac2 function and mtDNA (and thus proton pumping activity) leads to lethality. 

The presence of the *ANT1* ortholog gene in yeast gave the possibility to exploit yeast as a model system; in fact, studies on the pathogenic mechanism of *ANT1* mutations were mostly carried out in this organism. 

Homologous complementation studies in which the mutant allele was introduced into the null mutant allowed to analyze the effects of each pathogenic mutation on mitochondrial functions, for example through oxidative growth analysis, oxygen consumption measurements, respiratory cytochromes content quantification, ADP/ATP transport activity analyses [41,43,47,49]. In addition, for a non-conserved mutation, a y*AAC2*/h*ANT1* chimeric construct was used [44]. As the haploid yeast with nonfunctional Aac2 is *petite*-negative, a direct measurement of mtDNA instability is not possible. In an attempt to demonstrate a link between the pathological mutation A123D and mtDNA instability, Palmieri and colleagues proposed a viability test as an indirect measure, under the assumption that a lethal phenotype could result from deletion(s) or loss of mtDNA. Indeed, yeast expressing the equivalent *aac2^A137D^* mutant allele showed a very severe viability loss [41]. Interestingly, treatment with two well-known antioxidant drugs, dihydrolipoic acid and N-acetyl cysteine, partially rescued the viability decrease in the mutant strain, suggesting that oxidative stress could have a role in the pathogenesis of mtDNA damage [41].

However, since most mutations are dominant, the best model for studying these mutations must be considered as a strain carrying both a wild-type and mutant copy of the gene. Such a model was created by introducing mutations into a wild-type strain, thus mimicking human heterozygous condition, giving rise to heteroallelic haploid strains. The evaluation of these strains allowed clarifying some aspects, including the effect on mtDNA stability. For example, the phenotypic characterization of heteroallelic strains carrying *aac2^R96H^* or *aac2^R252G^* alleles not only served to demonstrate that the mutations are dominant, but also suggested that the dominance of R80H and R235G in *ANT1* is likely due to gain-of-function [48]. Notably, all the heteroallelic strains so far studied recapitulate the mtDNA instability found in patients, as demonstrated by an increase in the frequency of *petite* colonies [43,48].

Extensive studies were also carried out in yeast in an attempt to clarify the link between ANT1/Aac2 dysfunction and mtDNA instability, leading to different hypotheses. The ANT1 mutant variant may have a reduced activity in ATP/ADP translocation across the IMM that subsequently causes an imbalance in the mitochondrial deoxynucleotide pool. Consequently, it would affect the accuracy of mtDNA replication, thereby leading to the accumulation of mutant mtDNA [243]. Altered intramitochondrial ATP levels in the matrix due to reverse ADP/ATP exchange or a defect in nucleotide transport could cause electron transport stalling, increased ROS production, and consequent oxidative mtDNA damage [41]. Formation of an unregulated channel on the IMM, induced by a mutated form of Ant1, rather than a defect in ATP/ADP translocation, could be the primary pathogenic factor in human adPEO. Accumulation of mtDNA mutations may be a consequence of the loss of mtDNA replication precursors following membrane permeabilization [42]. More recently, it was proposed that misfolding and propensity to form large aggregates by Aac2 mutant could generate stress on the membrane, affecting mitochondrial biogenesis, particularly causing severe damage to the electron transport chain assembly and mtDNA integrity [45,251,252]. Nevertheless, the experiments performed so far are not yet conclusive, and none of these hypotheses seem to be totally clarifyied for now. It is not to be excluded that different mechanisms could coexist, giving a contribution to the pathophysiological mechanism.

## 4. Use of the Yeast Models for the Identification of Drugs by Means of a Drug Repurposing Approach

Currently, no approved treatments for mitochondrial diseases associated with mtDNA depletion and multiple deletions are available [253]. In recent years, yeast has been used as a model organism for the development of phenotypic screenings to identify molecules capable of suppressing mitochondrial disease-related phenotypes, including mtDNA instability. In 2011, an assay using the yeast *S. cerevisiae* to rapidly identify potential drugs that could be used to treat mitochondrial diseases was developed. This assay, called drug drop test, allows a high throughput analysis of chemical libraries in a reasonably short time. This approach exploits the respiratory-deficient phenotype of specific strains harboring mutations that affect the OXPHOS activity. The mutant strain is plated onto a medium containing a non-fermentable carbon source on which paper disks are placed; a different molecule is then deposited on each disk, using a disk for the negative control (the solvent in which the molecules are dissolved). The plate thus prepared is incubated at the temperature of interest and growth is monitored for several days. Since each drug diffuses from the disk forming a gradient, the great advantage of this technique is that it allows evaluating the effect of the tested molecules at different and continuous concentrations, avoiding the need to determine the effective and non-toxic dilutions. In particular, a molecule gives rise to a halo of growth around the filter if it is able to rescue the phenotype caused by the mutation harbored by the strain [254]. 

This approach can be used either to test chemical libraries containing novel drugs (natural and/or synthetic) or drugs previously approved by specific agencies such as the Food and Drug Administration (FDA) and the European Medicines Agency (EMA) for the treatment of other diseases. In the latter case, a drug repurposing approach, that is the investigation of existing drugs for new diseases, is used. This approach allows for a considerable reduction in times and costs, since the safety and pharmacological parameters have been already assessed [255]. It is based on the fact that several drugs have secondary targets or, alternatively, that the primary target inhibited by the drug participates in a pathway involved in the onset of the disease. Due to these characteristics, drug repurposing is an optimal approach, especially in the field of rare diseases, including mitochondrial ones [256]. 

Concerning mitochondrial diseases associated with mtDNA instability, the drug repurposing approach has been used to find drugs able to rescue the oxidative growth defects of *MIP1*, *MGM1*, *AAC2*, *SYM1*, and *RNR2* mutant strains. 

The *mip1^G651S^* mutant, carrying the mutation equivalent to *POLG* G848S, shows a defective oxidative growth compared to the wild-type *MIP1* at a non-permissive temperature (37 °C) due to the high instability of the mtDNA (*petite* frequency > 99%). Through the screening of ~1500 drugs, clofilium tosylate, an anti-arrhythmic drug, was found as a positive hit [257] able to decrease the *petite* frequency of a plethora of *mip1* mutant strains, to increase the protein levels of wild-type and mutant variants, and to partially restore the respiratory activity of the mutant strains. Further analyses showed that clofilium tosylate is able to rescue the phenotypes caused by mutations/deletions in *POLG* in the worm *Caenorhabditis elegans*, in zebrafish, and in patients’ fibroblasts, making it suitable as a potential treatment for *POLG*-associated pathologies [257,258].

The *mgm1^I322M^* mutant, harboring the hypomorphic mutation equivalent to I382M mutation in *OPA1*, shows a limited oxidative growth associated with both a strong defect in the maintenance of mtDNA (*petite* frequency > 95%) and a low respiratory activity (~5% compared to wild-type strain) at a non-permissive temperature (37° C). Through the screening of ~2600 drugs of two different chemical libraries, among which a library containing 1018 FDA-approved drugs and a library containing 1596 molecules with a high degree of chemical diversity, 42 positive hits were identified. Among these, 26 molecules were also able to partially recover the thermosensitive growth defect of a chimeric mutant strain harboring the *OPA1* S616L pathological mutation. Further analyses showed that six of them, able to decrease the mtDNA mutability in yeast, were able to improve the pathological phenotype of *opa1^−/−^* mouse embryo fibroblasts expressing the human *OPA1* isoform carrying two mutations, the R445H or D603H, associated with DOA plus and DOA, respectively. Interestingly, different drugs rescue different defects induced by mutations in *OPA1*, such as mitochondrial morphology, cell viability, or energetics, suggesting thatthe rescuing mechanisms of each drug are different. Analysis performed on DOA patients’ fibroblasts allowed to select the most promising molecule, tolfenamic acid, to be translated in a clinical trial for DOA or other neurodegeneration linked with *OPA1* mutations [259]. 

The haploid *aac2^M114P^* mutant, which carries the mutation equivalent to *ANT1* L98P found in adPEO patients, exhibits a defective respiratory growth, making it possible to search for molecules that restore this defect. The screening of 1018 FDA-approved compounds led to the identification of five positive hits, able to bring the level of oxygen consumption rate of the *aac2^M114P^* mutant strain to the wild-type level. Furthermore, these molecules were able to restore the respiratory activity and to reduce the mtDNA instability in the heteroallelic *AAC2*/*aac2^M114P^* strain, which mimics the human heterozygous condition of adPEO patients, and in the heteroallelic strain carrying the R96H mutation equivalent to the de novo dominant missense mutation R80H, associated with a more severe disease, thus expanding the possible applications for the treatment. Positive results on two drugs, albeit preliminary, were also obtained in *C. elegans*, indicating that these drugs identified in yeast are also beneficial in a multi-organ animal model and can be potentially applied to humans [260]. 

The thermosensitive yeast mutant *sym1^R51W^*, harboring the mutation equivalent to *MPV17* R50W, was exploited to identify potential therapeutic molecules for MDDS caused by mutations in *MPV17*. Through screening on a library containing 1018 FDA-approved drugs and on other six molecules previously identified as positive on another yeast model of MDDS, 10 drugs were found as positive hits, being able to rescue the OXPHOS growth defect and to reduce the mtDNA instability of the *sym1^R51W^* mutant strain. Rescue of the growth defect and a decrease in the *petite* frequency were also obtained in the absence of Sym1 protein, thus suggesting that the observed beneficial effect was due to a bypass mechanism. Further analysis showed that the decrease in mtDNA instability obtained with all ten drugs was associated with an increase in mitochondrial dNTP pools, especially of dTTP, providing evidence that the reduced availability of DNA synthesis precursors is the cause of the mtDNA maintenance defect in Sym1 deficiency. Some of these molecules were also able to reduce mtDNA instability on another MDDS yeast model, characterized by mutations in *MIP1* or *RNR2*, likely increasing the levels of dNTPs [40]. Although it is necessary to test these molecules on mammalian cells and model organisms, the identification of molecules capable of reducing the mtDNA instability in different MDDS yeast models makes them a starting point for developing drugs for the treatment of diseases caused by mutations in different genes but all resulting in mtDNA synthesis defects.

## 5. Conclusions

In the last ten years, most of the novel pathological mutations were found through whole-exome sequencing or through whole-genome sequencing. Sometimes, only the exome or the genome of the proband are sequenced, and, besides the pathological mutations, other mutations are often identified. It is thus fundamental to evaluate whether the putative pathological mutation is the cause of the disease or not, and the use of specific models in which just a single mutation is introduced, such as yeast, can be helpful. Because of the conservation of genes and pathways during evolution, the study of human genetic diseases associated with mtDNA depletion and multiple deletions has also been directly addressed in the model organism *Saccharomyces cerevisiae*. Thanks to the use of yeast as a model system, the causal relationship between pathologies associated with mtDNA instability and novel nuclear mutations has been established or confirmed for tens of mutations. Besides validation, yeast has been also used to determine the pathogenic mechanisms behind these mutations. In this regard, it must be underlined that yeast also has some limitations, among which a different size of the mtDNA, partial differences in the replication process, and the fact that, if two mtDNA molecules are present, the patients are mainly heteroplasmic, whereas yeast is homoplasmic. Despite these differences, pathological mechanisms identified in yeast have been observed in mutant cells derived from patients or in biochemical assays (reviewed in [261]).

To our knowledge, mutations in approximately 25 nuclear genes are associated with diseases characterized by depletion and/or multiple deletions. More than 15 genes are conserved in yeast, although, for some of them, such as *TFAM*/*ABF2* or *SSBP1*/*RIM1*, the protein similarity is low. Mutations in six genes have been validated in yeast, mainly through homologous complementation (*ANT1/AAC2, MRM2/MRM2, MPV71/SYM1, POLG/MIP1, RRM2B/RNR2*) or, to a lesser extent, through chimeric complementation (*ANT1/AAC2, OPA1/MGM1, POLG/MIP1*). 

A major challenge regarding mitochondrial diseases associated with mtDNA depletion and multiple deletions is the availability of pharmacological treatments. For some of these diseases, preclinical studies have been performed or are ongoing [262,263,264,265,266,267]. Recently, an open-label clinical study showed that the administration of deoxynucleoside monophosphates and deoxynucleosides to 16 children affected by *TK2*-related disease improved, in most cases, the health condition [268]. However, the identification of therapeutic molecules effective on a broad spectrum of mitochondrial pathologies would be critical. From this point of view, yeast disease models have proven to be useful, thanks to the existence of numerous disease models and to the technique reported above, which allows for the analysis of many molecules in short time. 

## Figures and Tables

**Table 1:** Genes associated with mitochondrial diseases characterized by mtDNA depletion and/or multiple deletions.

Human Gene	Protein Function	Disease	OMIM Number	Onset	Inheritance	mtDNA Alteration	Main Phenotype	Yeast Gene	Study in Yeast
*ABAT*	4-aminobutyrate aminotransferase	GABA-transaminase deficiency	613163	Infancy	AR	Multiple deletions	Encephalopathy, myopathy, and elevated GABA	*UGA1*	/
*AGK*	Acylglycerol kinase	MDDS 10, Sengers syndrome	212350	Neonatal	AR	Depletion	Cardiac and skeletal myopathy and cataract	NP	/
*DGUOK*	Mitochondrial deoxyguanosine kinase	MDDS 3	251880	Neonatal period, infancy, or childhood	AR	Depletion	Hepatopathy and encephalopathy	NP	/
		PEO, autosomal recessive 4	617070	Early or mid-adulthood	AR	Multiple deletions	Myopathy and ophthalmoplegia		
*DNA2*	DNA replication helicase/nuclease 2	PEO, autosomal dominant 6	615156	Childhood or early adulthood	AD	Multiple deletions	Myopathy and ophthalmoplegia	*DNA2*	/
*FBXL4*	F-box and leucine-rich repeat protein 4	MDDS 13	615471	Neonatal period or infancy	AR	Depletion	Encephalopathy and myopathy	NP	/
*LIG3*	Ligase III	Neurogastrointestinal encephalomyopathy		Infancy to adolescence	AR	Depletion	Gut dysmotility, encephalopathy, and myopathy	NP	/
*MFN2*	Mitofusin 2	Hereditary motor and sensory neuropathy VIA; DOA	601152	Early childhood	AD	Multiple deletions	Optic atrophy and neuropathy	*FZO1*	/
*MGME1*	Mitochondrial exonuclease 1	MDDS 11	615084	Childhood or early adulthood	AR	Depletion and multiple deletions	Myopathy	NP	/
*MPV17*	IMM protein	PEO, autosomal recessive		Adulthood	AR	Multiple deletions	Ophthalmoplegia leukoencephalopathy and/or parkinsonism	*SYM1*	[16,17]
		Neuromyopathic MDMD		Adulthood	AR	Multiple deletions	Neuropathy and myopathy		
		MDDS 6	256810	Neonatal period, infancy, or early childhood	AR	Depletion	Neuropathy, hepatopathy and/or encephalopathy		
*MRM2*	Mitochondrial ribosomal RNA methyltransferase 2	MDDS 17	618567	Infancy	AR	Depletion	MELAS-like with encephalopathy, lactic acidosis and stroke-like episodes	*MRM2*	[18]
*OPA1*	Mitochondrial dynamin-like GTPase	MDDS 14	616896	Neonatal or infancy	AR	Depletion	Cardiomyopathy, encephalopathy	*MGM1*	[19,20,21]
		DOA	165500	Childhood or early adulthood	AD	(Multiple deletions)	Optic atrophy		
		DOA plus	125250	Childhood or early adulthood	AD	Multiple deletions	Optic atrophy with deafness, ophthalmoplegia, myopathy, ataxia, and/or neuropathy		
*POLG*	DNA polymerase γ	Childhood myocerebrohepatopathy spectrum disorders		Infancy	AR	Depletion	Hypotonia, hepatopathy, developmental delay	*MIP1*	[22,23,24,25,26,27,28,29,30,31,32,33,34,35,36,37,38,39]
		MDDS 4A	203700	Early childhood	AR	Depletion	Alpers–Huttenlocher syndrome with encephalopathy, neuropathy, and hepatopathy		
		MDDS 4B	613662	Childhood to adulthood	AR	Depletion and multiple deletions	MNGIE with gastrointestinal dysmotility, myopathy, and neuropathy		
		Mitochondrial recessive ataxia syndrome	607459	Adolescence, early adulthood	AR	Multiple deletions	SANDO/SCAE, ANS, MEMSA with ataxia, neuropathy, encephalopathy, epilepsy and/or myopathy		
		PEO, autosomal dominant 1	157640	Adulthood	AD	Multiple deletions	Ophthalmoplegia and myopathy		
		PEO, autosomal recessive	258450	Adolescence, adulthood	AR	Multiple deletions	Ophthalmoplegia		
*POLG2*	DNA polymerase γ accessory subunit	MDDS 16 (hepatic type)	618528	Infancy	AR	Depletion	Hepatophty	NP	/
		MDDS 16B	619425	Childood	AR	Depletion	Neuroophthalmic type		
		PEO, autosomal dominant 4	610131	Infancy to adulthood	AD	Multiple deletions	Myopathy and ophthalmoplegia		
*RNASEH1*	Ribonuclease H1	PEO, autosomal recessive 2	616479	Adulthood	AR	Multiple deletions	Ophthalmoplegia	*RNH1*	/
*RRM2B*	Ribonucleotide reductase, M2 B	MDDS 8A and 8B	612075	Infancy	AR	Depletion	Myopathy, encephalopathy and tubulopathy or MNGIE	*RNR2*	[40]
		PEO, autosomal recessive		Childhood	AR	Multiple deletions	Ophthalmoplegia and myopathy		
		PEO, autosomal dominant 5	613077	Adulthood	AD	Multiple deletions	Ophthalmoplegia and myopathy		
*SLC25A21*	Mitochondrial oxodicarboxylate carrier	MDDS 18	618811	Early childhood	AR	Depletion	Muscular atrophy and myopathy	*ODC1/* *ODC2*	/
*SLC25A4* (*ANT1*)	Mitochondrial ADP/ATP translocator	MDDS 12A (cardiomyopathic type)	617184	Neonatal	AD	Depletion	Myopathy and cardiomyopathy	*AAC2* (*AAC1*, *AAC3*)	[41,42,43,44,45,46,47,48,49]
		MDDS 12B (cardiomyopathic type)	615418	Childhood	AR	Depletion and multiple deletions	Myopathy and cardiomyopathy		
		PEO, autosomal dominant 2	609283	Adulthood	AD	Multiple deletions	Ophthalmoplegia and myopathy		
*SLC25A10* (*DIC*)	Mitochondrial dicarboxylate carrier	MDDS 19	618972	Infancy	AR	Depletion	Encephalopathy an hypotonia	*DIC1*	/
*SSBP1*	Single-stranded DNA-binding protein 1	Optic atrophy 13	165510	Infancy to early adulthood	AD	Depletion	Optic atrophy	*RIM1*	/
*SUCLA2*	Succinyl-CoA ligase, β subunit	MDDS 5	612073	Infancy or early childhood	AR	Depletion	Encephalopathy and myopathy with or without methylmalonic aciduria	*LSC2*	/
*SUCLG1*	Succinyl-CoA ligase, α subunit	MDDS 9	245400	Neonatal period or infancy	AR	Depletion	Encephalopathy and myopathy with methylmalonic aciduria	*LSC1*	/
*TFAM*	Mitochondrial transcription factor 1	MDDS 15	617156	Neonatal	AR	Depletion	Hepatocerebral syndrome	*ABF2*	/
*TOP3A*	DNA topoisomerase III	PEO, autosomal recessive 5	618098	Adulthood	AR	Multiple deletions	Ophthalmoplegia and ataxia	*TOP3*	/
*TK2*	Mitochondrial thymidine kinase	PEO, autosomal recessive 3	617069	Mid-Adulthood	AR	Multiple deletions	Ophthalmoplegia and myopathy	NP	/
		MDDS 2	609560	Infancy or childhood	AR	Depletion	Myopathy,		
*TWNK*	Twinkle mtDNA helicase	MDDS 7 (hepatocerebral type), IOSCA	271245	Infancy	AR	Depletion	Ataxia, encephalopathy, and neuropathy	NP	/
		PEO, autosomal dominant 3	609286	Early adulthood	AD	Multiple deletions	Ophthalmoplegia and myopathy		
		Hepatocerebral MDMD		Neonatal or early infancy	AR	Depletion	Alpers-like with encephalopathy and hepatopathy		
*TYMP*	Thymidine phosphorylase	MDDS 1	603041	Adolescence to adulthood	AR	Depletion and multiple deletions	MNGIE with gastrointestinal dysmotility, myopathy, and neuropathy	NP	/

Genes are reported if mtDNA depletion and/or mtDNA deletions are found in most affected patients. Pathologies are reported only if mtDNA depletion and/or deletions occur. Yeast studies are reported only if yeast was used to confirm the pathogenicity of the mutations found in patients. Abbreviations: PEO: progressive external ophthalmoplegia; DOA: dominant optic atrophy; MDMD: mitochondrial DNA maintenance defects; MDDS: Mitochondrial DNA depletion syndrome; NP: not present.
